# Validity of claims‐based algorithms for selected cancers in Japan: Results from the VALIDATE‐J study

**DOI:** 10.1002/pds.5263

**Published:** 2021-06-01

**Authors:** Cynthia de Luise, Naonobu Sugiyama, Toshitaka Morishima, Takakazu Higuchi, Kayoko Katayama, Sho Nakamura, Haoqian Chen, Edward Nonnenmacher, Ryota Hase, Sadao Jinno, Mitsuyo Kinjo, Daisuke Suzuki, Yoshiya Tanaka, Soko Setoguchi

**Affiliations:** ^1^ Safety Surveillance Research Pfizer Inc New York New York USA; ^2^ Inflammation & Immunology, Medical Affairs Pfizer Japan Tokyo Japan; ^3^ Department of Cancer Strategy, Cancer Control Center Osaka International Cancer Institute Osaka Japan; ^4^ Blood Transfusion Department Dokkyo Medical University Saitama Medical Center Koshigaya Japan; ^5^ Cancer Prevention and Cancer Control Division Kanagawa Cancer Center Research Institute Yokohama Japan; ^6^ School of Health Innovation Kanagawa University of Human Services Yokosuka Japan; ^7^ Department of Clinical Oncology Faculty of Medicine, Yamagata University Yamagata Japan; ^8^ Center for Pharmacoepidemiology and Treatment Science Rutgers Institute for Health, Health Care Policy and Aging Research New Brunswick New Jersey USA; ^9^ Department of Infectious Diseases Kameda Medical Center Kamogawa Japan; ^10^ Department of Infectious Diseases Japanese Red Cross Narita Hospital Narita Japan; ^11^ Section of Rheumatology Kobe University School of Medicine Kobe Japan; ^12^ Division of Rheumatology Okinawa Chubu Hospital Uruma Japan; ^13^ Department of Infectious Diseases Fujita Health University Toyoake Japan; ^14^ The First Department of Internal Medicine School of Medicine, University of Occupational and Environmental Health Japan Kitakyushu Japan; ^15^ Department of Medicine Rutgers Robert Wood Johnson Medical School and Institute for Health New Brunswick New Jersey USA

**Keywords:** cancer, claims‐based algorithms, Japan, positive predictive value, sensitivity, specificity, validation

## Abstract

**Purpose:**

Real‐world data from large administrative claims databases in Japan have recently become available, but limited evidence exists to support their validity. VALIDATE‐J validated claims‐based algorithms for selected cancers in Japan.

**Methods:**

VALIDATE‐J was a multicenter, cross‐sectional, retrospective study. Disease‐identifying algorithms were used to identify cancers diagnosed between January or March 2012 and December 2016 using claims data from two hospitals in Japan. Positive predictive values (PPVs), specificity, and sensitivity were calculated for prevalent (regardless of baseline cancer‐free period) and incident (12‐month cancer‐free period; with claims and registry periods in the same month) cases, using hospital cancer registry data as gold standard.

**Results:**

22 108 cancers were identified in the hospital claims databases. PPVs (number of registry cases) for prevalent/incident cases were: any malignancy 79.0% (25 934)/73.1% (18 119); colorectal 84.4% (3519)/65.6% (2340); gastric 87.4% (3534)/76.8% (2279); lung 88.1% (2066)/79.9% (1636); breast 86.4% (4959)/59.9% (3185); pancreatic 87.1% (582)/80.4% (508); melanoma 48.7% (46)/42.9% (36); and lymphoma 83.6% (1457)/77.8% (1035). Specificity ranged from 98.3% to 100% (prevalent)/99.5% to 100% (incident); sensitivity ranged from 39.1% to 67.6% (prevalent)/12.5% to 31.4% (incident). PPVs of claims‐based algorithms for several cancers in patients ≥66 years of age were slightly higher than those in a US Medicare population.

**Conclusions:**

VALIDATE‐J demonstrated high specificity and modest‐to‐moderate sensitivity for claims‐based algorithms of most malignancies using Japanese claims data. Use of claims‐based algorithms will enable identification of patient populations from claims databases, while avoiding direct patient identification. Further research is needed to confirm the generalizability of our results and applicability to specific subgroups of patient populations.

**Key Points**

- Real‐world data from healthcare claims databases are available in Japan, but few validation studies exist to support the validity of these data.
- In VALIDATE‐J, a multicenter validation study in Japan, algorithms were developed from institutional claims data and validated against hospital‐based cancer registry data.
- Positive predictive value (PPV), specificity, and sensitivity were computed for incident and prevalent cancers; PPVs for several cancers were higher than those reported in the USA.
- One of the first and largest validation studies in Japan, VALIDATE‐J will inform future claims‐based research and serve as a model for validation studies for claims‐based post‐marketing studies in Japan.

## 1 INTRODUCTION

The incidence of many primary cancers is increasing in Japan, with colorectal, lung, and gastric cancers as the most frequently reported cancer types in 2018.^1^ Cancer remains a leading cause of mortality in Japan.^2^

Real‐world data are essential for understanding cancer epidemiology, both in the general population and as part of drug safety surveillance. Administrative healthcare claims databases provide a valuable source of longitudinal, real‐world data for pharmacoepidemiology, comparative effectiveness research, and health services/outcome research. Claims‐based definitions for cancers have been developed and validated in the USA and EU for the identification of incident cases of breast, lung, gastric, colorectal, and hematologic cancers in hospital or commercial administrative databases^3^, ^4^, ^5^, ^6^ and, in the USA, for determining the incidence of cancer among patients with inflammatory diseases receiving tumor necrosis factor inhibitors.^7^, ^8^

Multiple claims databases, such as the National Database of Health Insurance Claims and Specific Health Checkups of Japan, the Japan Medical Data Center, and the Medical Data Vision, are now available to academic and industry researchers in Japan. The Pharmaceuticals and Medical Devices Agency encourages the use of claims‐based pharmacoepidemiology research for drug safety surveillance in Japan, and requires validation studies to support the credibility of claims database research for post‐marketing surveillance.^9^ However, validation studies of Japanese claims data are still limited, as evidenced by a review of claims‐based validation studies in the Asia‐Pacific region^10^, ^11^ and other published studies.^12^, ^13^, ^14^, ^15^ Given the unique features of claims data and the clinical practice environment in Japan, validated claims‐based algorithms developed in other regions (eg, USA or Europe) are unlikely to be relevant to claims database research in Japan.

The Validity of Algorithms in Large Databases: Infectious Diseases, Rheumatoid Arthritis, and Tumor Evaluation in Japan (VALIDATE‐J) study investigated the validity of several predefined disease‐identifying algorithms using hospital claims data in Japan. Here, we report the positive predictive value (PPV), specificity, and sensitivity of claims‐based algorithms for selected malignancies (any malignancy, colorectal, gastric, lung, breast, pancreatic, melanoma, and lymphoma) from VALIDATE‐J.

## 2 METHODS

### 2.1 Study design

**FIGURE 1:**
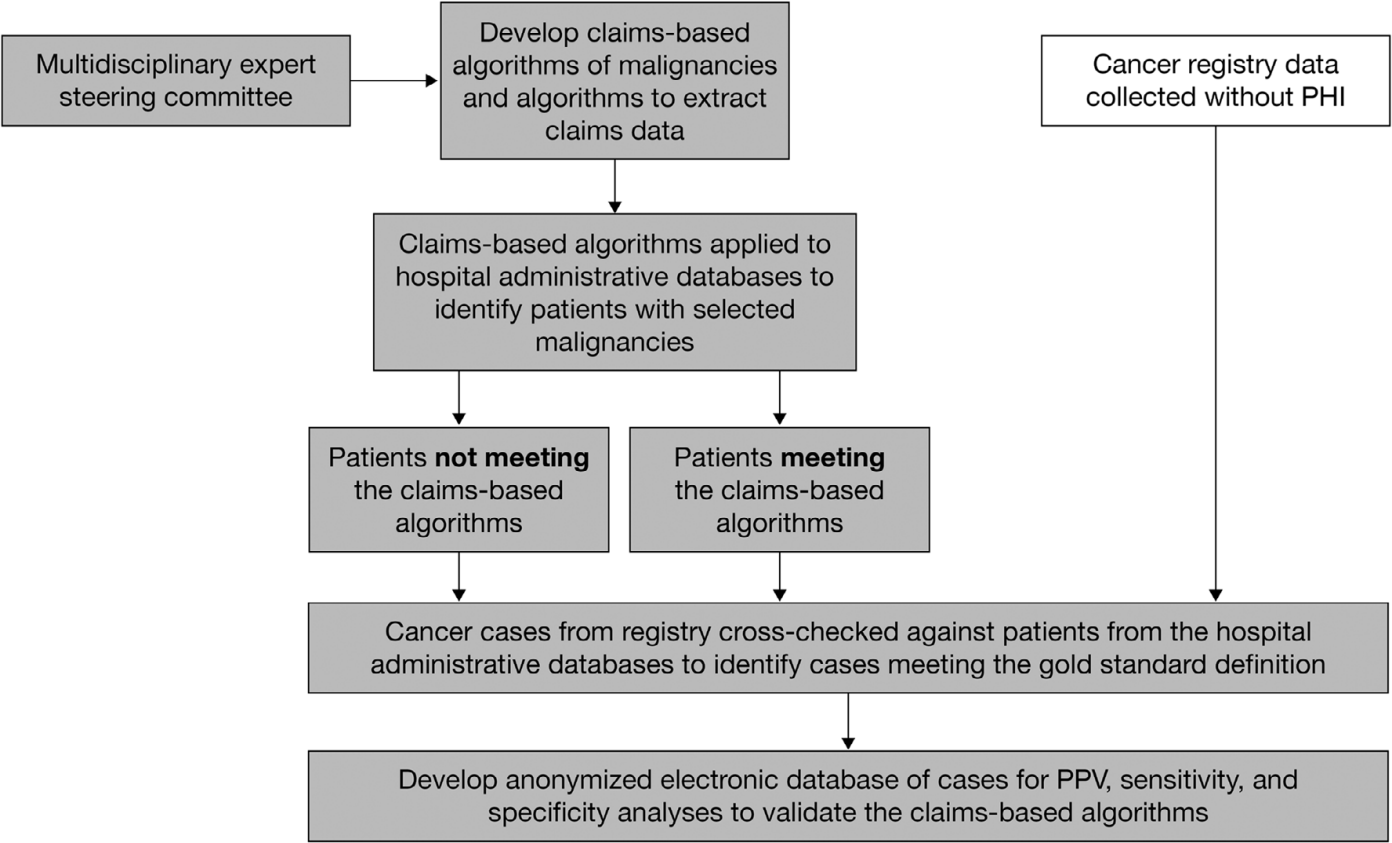
Study flow chart. PHI, Protected Health Information; PPV, positive predictive value

VALIDATE‐J was a cross‐sectional, retrospective study of claims data, medical records, and cancer registry data from two general acute‐care hospitals in Chiba, Japan – a large 917‐bed private teaching and cancer care hospital designated by the national government, located in a rural area (Hospital A), and a large 716‐bed community teaching and cancer care hospital designated by the local government, located in a city area (Hospital B) – conducted between December 2017 and February 2019. The overall study objectives were to validate claims‐based algorithms for rheumatoid arthritis (RA), infectious diseases, and malignancies. Data for RA and infectious disease will be reported elsewhere. An overview of the study is shown in Figure 1.

Prior to study initiation, claims‐based algorithms for any malignancy, colorectal, gastric, lung, breast, and pancreatic cancers, melanoma, and lymphoma, were developed and modified by a steering committee of experts in oncology, Japanese cancer registries, and epidemiology (Table 1). The algorithms were based, in part, on previously tested definitions,^6^ and were modified to reflect the Japanese clinical practice environment and the coding rules and practices for claims unique to Japan. Algorithms based on combinations of International Classification of Diseases (ICD) diagnosis codes for each selected malignancy type and drug, and procedure codes for relevant therapies, were developed and modified in a pilot phase of the study and subsequently used to identify cases in the two hospital databases. A confirmed cancer diagnosis in the cancer registry at either hospital between January 01, 2012 and December 31, 2016 was defined as the gold standard. Cancer diagnoses in the hospital cancer registry were defined by ICD for Oncology, 3rd Edition (ICD‐O‐3) topographical and morphological codes. The original ICD‐O‐3 codes were converted to ICD‐10 codes according to the International Association of Cancer Registries CanReg Tools v2.^16^ A list of the ICD‐10 diagnosis codes used is provided in Table S1.

**TABLE 1:** Claims‐based algorithms and gold standard definitions for selected malignancy types

Malignancy type	Claims‐based criteria	Gold standard
Colorectal, gastric, lung, breast,^a^ pancreatic, melanoma, and lymphoma^a^ ^,^ ^b^	One diagnosis within a claim‐month AND Chemotherapy and/or radiation and/or surgery within same claim‐month or ±1 claim‐month	Confirmation of cancer diagnosis defined by a specific ICD‐10 code in the cancer registry at either hospital^c^ ^,^ ^d^
“Any malignancy”	One diagnosis within a claim‐month AND A procedure record of cancer management within same claim‐month or ±1 claim‐month	

Abbreviations: IACR, International Association of Cancer Registries; ICD‐10, International Classification of Diseases, 10th Edition.

Claim‐month implies that the codes were searched during a 3‐month period, including the same calendar month of the diagnosis as well as 1 month before or after the month of the cancer diagnosis.

^a^ Patients with breast cancer or lymphoma prescribed methotrexate 2.5 mg were required to have a prescription volume for the claim‐month exceeding 100 mg.

^b^ Criteria for lymphoma do not include surgery.

^c^ A list of the ICD‐10 diagnosis codes used is provided in Table S1.

^d^ ICD‐O‐3 codes were converted to ICD‐10 codes according to IACR CanReg Tools v2.^16^

An Independent Ethics Committee and the Institutional Review Board at each participating hospital approved the study protocol. The study was conducted in accordance with accepted practices for pharmacoepidemiology studies issued by the International Society for Pharmacoepidemiology and the Council for International Organizations of Medical Sciences. Patients identified in the claims databases were not required to provide consent and could opt out from participating in the study.

### 2.2 Study cohort

The claims‐based cohort included patients treated as outpatients or inpatients at either hospital between January 01, 2012 (Hospital A) or March 01, 2012 (Hospital B) and December 31, 2016, who met the prespecified claims‐based criteria for each selected malignancy type (Table 1). Cases were defined as “prevalent”, that is, regardless of baseline‐free cancer period, or “incident”, that is, cases with a 12‐month cancer‐free period prior to case ascertainment (primary algorithm of incident cases). The ICD‐10 diagnosis codes (Table S1) were converted from ICD‐O‐3 codes and used to determine the different cancer subpopulations. Duplications were then identified using patient IDs and removed from the prevalence population. The incidence population was derived from the previously de‐duplicated prevalence population. To benchmark how the algorithms performed, the results of validity measures in a subset of patients ≥66 years of age were compared to those in a validation study of US patients ≥65 years of age identified from the Medicare/Pennsylvania Assistance Contract for the Elderly program data linked with the state cancer registry (1997–2000).^6^ State cancer registry data were used as gold standard for validation of the US data.

### 2.3 Validity measures

PPV, specificity, and sensitivity of claims‐based algorithms for each malignancy type were calculated using the registry‐based gold‐standard diagnosis, based on prevalent and incident cases, with claim and registry periods within the same month.

### 2.4 Statistical analysis

Demographics and disease characteristics were summarized using descriptive statistics (means and standard deviations for continuous variables, and percentages and counts for dichotomous variables).

PPV for each claims‐based algorithm was calculated as the number of cases meeting the claims‐based algorithm that were confirmed in the cancer registry (ie, true positives) divided by the total number of cases meeting the claims‐based algorithm (ie, true and false positives) (Table S2). Specificity was calculated as the number of cases that did not meet the claims‐based algorithm and were not found in the registry (ie, true negatives) divided by the subset of cases from both hospitals that were not in the linked cancer registry, regardless of whether they met the claims‐based algorithms (ie, true negative and false positive cases) (Table S2). Sensitivity was calculated as the number of true positive cases divided by the total number of confirmed cases in the linked hospital cancer registry (ie, true positive and false negative cases) (Table S2). As a sensitivity analysis, incident cases were also measured with a 6‐month cancer‐free period prior to case ascertainment (in addition to a 12‐month cancer‐free period).

Calculations of specificity and sensitivity were based on the following assumptions: case identification in the cancer registry was close to 100%, and all the data from the hospital cancer registries could be linked to hospital claims data. 95% confidence intervals (CI) for PPV, specificity, and sensitivity were calculated using the normal approximation of the binomial distribution. Deidentified data were analyzed using Python version 3.6.0 (2016).

## 3 RESULTS

### 3.1 Patients

During the ascertainment period (2012–2016), a total of 25 934 cases of malignancies specified in this study were recorded in the hospital cancer registries.

Demographics and disease characteristics for cases identified in the hospital cancer registries are shown in Table 2 and Table S3 (data for individual hospitals are shown in Tables S4, S5). Mean age was 64.8 years for patients with any malignancy and ≥65 years for colorectal, gastric, lung, and pancreatic cancers, melanoma and lymphoma, except for breast cancer (mean age was 55.6 years) for which 65% of patients were 40–64 years of age. Approximately half of the malignancies were in females, except for breast cancers which were all in females for this analysis, and colorectal, gastric, and lung cancers for which most cases were in males. Histology from biopsy or resection specimen was the most common diagnostic method for all cancer types and was used for >92% of all malignancies. *In situ* cancer was the most common type of breast cancer (21%) identified, and localized cancer was most common among gastric cancer, accounting for 66% of cases. A numerically higher proportion of patients with lung and pancreatic cancers, or lymphoma had Stage IV disease compared with other cancer types. Approximately one‐third of all cases (any malignancy) had been treated with surgery or chemotherapy.

**TABLE 2:** Demographics and disease characteristics of cases from the cancer registries at two hospitals

	Any malignancy *N* = 25 934	Colorectal *N* = 3519	Gastric *N* = 3534	Lung *N* = 2066	Breast *N* = 4959	Pancreatic *N* = 582	Melanoma *N* = 46	Lymphoma *N* = 1457
Age (years), mean (SD)	64.8 (22.7)	68.0 (11.2)	70.3 (10.2)	69.9 (45.2)	55.6 (12.8)	70.8 (10.7)	70.9 (14.1)	66.2 (14.6)
Female, *n* (%)	12 864 (49.6)	1391 (39.5)	973 (27.5)	687 (33.3)	4959 (100.0)	260 (44.7)	23 (50.0)	664 (45.6)
Method of diagnosis, *n* (%)								
Histology	23 969 (92.4)	3408 (96.8)	3481 (98.5)	1671 (80.9)	4890 (98.6)	349 (60.0)	40 (87.0)	1368 (93.9)
Cytology	307 (1.2)	10 (0.3)	6 (0.2)	92 (4.5)	33 (0.7)	18 (3.1)	0 (0)	22 (1.5)
Pathology^a^	2434 (9.4)	283 (8.0)	278 (7.9)	335 (16.2)	511 (10.3)	61 (10.5)	5 (10.9)	130 (8.9)
Tumor marker	140 (0.5)	7 (0.2)	1 (<0.1)	9 (0.4)	2 (<0.1)	31 (5.3)	0 (0)	4 (0.3)
Direct visualization	451 (1.7)	50 (1.4)	20 (0.6)	51 (2.5)	3 (<0.1)	79 (13.6)	4 (8.7)	6 (0.4)
Radiology	639 (2.5)	25 (0.7)	12 (0.3)	167 (8.1)	6 (0.1)	74 (12.7)	1 (2.2)	6 (0.4)
Clinical diagnosis	18 (0.1)	1 (<0.1)	0 (0)	1 (<0.1)	0 (0)	2 (0.3)	0 (0)	1 (0.1)
Unknown/missing	122 (0.5)	12 (0.3)	5 (0.1)	9 (0.4)	5 (0.1)	9 (1.5)	0 (0)	15 (1.0)
Treatment, *n* (%)								
Surgical	8556 (33.0)	1235 (35.1)	1158 (32.8)	586 (28.4)	1577 (31.8)	160 (27.5)	30 (65.2)	53 (3.6)
Celoscopic	4310 (16.6)	1082 (30.7)	368 (10.4)	244 (11.8)	2321 (46.8)	9 (1.5)	46 (100.0)	4 (0.3)
Endoscopic	3140 (12.1)	792 (22.5)	1419 (40.2)	3 (0.1)	2 (<0.1)	58 (10.0)	46 (100.0)	4 (0.3)
Radiation therapy	3398 (13.1)	161 (4.6)	15 (0.4)	489 (23.7)	1316 (26.5)	26 (4.5)	1 (2.2)	164 (11.3)
Chemotherapy	7448 (28.7)	1061 (30.2)	1061 (30.0)	775 (37.5)	1331 (26.8)	268 (46.0)	6 (13.0)	1083 (74.3)
Immunotherapy	266 (1.0)	5 (0.1)	4 (0.1)	33 (1.6)	54 (1.1)	1 (0.2)	2 (4.3)	1 (0.1)
*N* missing	1282	186	157	116	108	52	2	87
Endocrine therapy	1362 (5.3)	42 (1.2)	17 (0.5)	78 (3.8)	475 (9.6)	19 (3.3)	46 (100.0)	14 (1.0)
*N* missing	2	0	0	0	1	0	0	0
CCI, mean (SD)^b^	2.1 (1.2)	2.1 (1.2)	2.5 (1.1)	2.4 (1.0)	1.8 (1.0)	2.2 (0.8)	1.9 (1.3)	2.2 (0.7)

Abbreviations: CCI, Charlson Comorbidity Index; *N*, total number of patients; *n*, number of patients in each category; SD, standard deviation.

^a^ Pathology indicates either histological or cytological diagnoses.

^b^ CCI was calculated by assigning comorbidity scores^17^ to patients within each separate cancer pool. The mean score was calculated for each patient by summing their scores and dividing them by the number of scores they had. All patients' average scores were then pooled and divided by the population size of each corresponding cancer category.

### 3.2 Claims‐based cases

A total of 22 108 prevalent cases of malignancies were identified in the hospital claims databases using the prespecified claims‐based algorithms (Hospital A, *n* = 14 257; Hospital B, *n* = 7851). The number of cases by cancer type was: any malignancy (*n* = 22 108), colorectal (*n* = 1717), gastric (*n* = 1784), lung (*n* = 1135), breast (*n* = 3880), pancreas (*n* = 303), melanoma (*n* = 37), and lymphoma (*n* = 965) (Table 3). For both claims‐based and registry cases, the most common malignancy was breast cancer (claims‐based, *n* = 3880; registry‐based, *n* = 4959) and the least common was melanoma (claims‐based, *n* = 37; registry‐based, *n* = 46).

**TABLE 3:** PPV, specificity, and sensitivity of the claims‐based algorithms for selected malignancies vs gold standard cancer diagnosis (both hospitals; prevalent cases)

Malignancy	N_C_/N_R_	PPV, % (95% CI)	Specificity, % (95% CI)	Sensitivity, % (95% CI)
Any malignancy	22 108/25 934^a^	78.95 (78.41–79.49)	98.33 (98.28–98.38)	67.30 (66.73–67.87)
Colorectal	1717/3519^b^	84.39 (82.67–86.11)	99.91 (99.90–99.92)	41.18 (39.55–42.80)
Gastric	1784/3534^c^	87.44 (85.91–88.98)	99.93 (99.92–99.94)	44.14 (42.51–45.78)
Lung	1135/2066^d^	88.11 (86.22–89.99)	99.96 (99.95–99.96)	48.40 (46.25–50.56)
Breast	3880/4959^e^	86.42 (85.34–87.50)	99.82 (99.81–99.84)	67.61 (66.31–68.92)
Pancreatic	303/582	87.13 (83.36–90.90)	99.99 (99.98–99.99)	45.36 (41.32–49.41)
Melanoma	37/46	48.65 (32.54–64.75)	99.99 (99.99–100.0)	39.13 (25.03–53.23)
Lymphoma	965/1457^f^	83.63 (81.29–85.96)	99.95 (99.94–99.96)	55.39 (52.84–57.94)

Abbreviations: CI, confidence interval; N_C_, number of claims‐based cases; N_R_, number of registry cases; *n*, duplicate cases; PPV, positive predictive value.

*Note*: Number of duplicate cases removed for the analysis.

^a^*n* = 2901.

^b^*n* = 227.

^c^*n* = 205.

^d^*n* = 79.

^e^*n* = 226 plus 37 males removed.

^f^*n* = 10.

### 3.3 Validity of claims‐based cancer algorithms

#### 3.3.1 Prevalent cases

PPV for prevalent cases was nearly 80% for any malignancy, and was lowest for melanoma and highest for lung cancer (Table 3). Specificity was 98% for any malignancy and nearly 100% for all selected malignancies. Sensitivity was lower than specificity for any malignancy; it was lowest for melanoma and highest for breast cancer (Table 3).

#### 3.3.2 Incident cases

For incident cases with a 12‐month cancer‐free period and with claims and registry periods within the same month, PPV for any malignancy was nearly 75%, and was lowest for melanoma and highest for pancreatic cancer (Table 4). Specificity was nearly 100% for any malignancy and for all selected malignancies (Table 4). Sensitivity was substantially lower and was <25% for any malignancy (lowest for breast cancer and highest for lymphoma) (Table 4). Sensitivity analyses using a 6‐month cancer‐free period vs a 12‐month cancer‐free period produced similar validity measures (data not shown).

**TABLE 4:** PPV, specificity, and sensitivity of the claims‐based algorithms for selected malignancies vs gold standard cancer diagnosis (both hospitals; incident cases^a^)

Malignancy	N_R_	PPV, % (95% CI)	Specificity, % (95% CI)	Sensitivity, % (95% CI)
Any malignancy	18 119	73.08 (71.94–74.22)	99.45 (99.43–99.48)	23.39 (22.77–24.01)
Colorectal	2340	65.60 (62.14–69.07)	99.92 (99.91–99.93)	20.21 (18.59–21.84)
Gastric	2279	76.83 (73.60–80.06)	99.95 (99.94–99.96)	22.11 (20.41–23.82)
Lung	1636	79.87 (76.74–83.00)	99.96 (99.95–99.97)	30.81 (28.57–33.04)
Breast	3185	59.94 (56.21–63.67)	99.91 (99.90–99.92)	12.50 (11.35–13.64)
Pancreatic	508	80.42 (73.92–86.92)	99.99 (99.99–99.99)	22.64 (19.00–26.28)
Melanoma	36	42.86 (21.69–64.02)	100.00 (99.99–100.00)	25.00 (10.85–39.15)
Lymphoma	1035	77.75 (73.76–81.74)	99.97 (99.96–99.98)	31.40 (28.57–34.23)

Abbreviations: CI, confidence interval; N_R_, number of registry cases; PPV, positive predictive value.

^a^ Incident cases represent those with a 12‐month cancer‐free period and with claims and registry periods in the same month.

An alternative algorithm for “any malignancy” requiring two cancer diagnoses with the same first three digits of the ICD‐codes, within the same claim‐month or ±1 claim‐month, was tested. This algorithm performed sub‐optimally in terms of PPV, specificity, and sensitivity, compared with the primary algorithm for “any malignancy” (Table S6).

#### 3.3.3 Measurements between hospitals

For prevalent cases from individual hospitals (Tables S7, S8), PPVs were consistently higher, by generally ≥10%, in Hospital A for all cancers, except melanoma, for which the PPV was higher for cases from Hospital B, and lymphoma, for which PPVs were similar between hospitals. Sensitivity measures varied, with no discernable pattern between hospitals.

For incident cases from individual hospitals (Tables S9, S10), PPVs were ≥10% higher for gastric, lung, breast, and pancreatic cancers from Hospital A, and 28% higher for melanoma and 6% higher for lymphoma from Hospital B. As with prevalent cases, sensitivity measures for incidence cases between hospitals followed no discernable pattern.

#### 3.3.4 Benchmarking results in Japanese patients ≥66 years of age and a US Medicare population

Japanese claims data showed that PPV, specificity, and sensitivity for colorectal, gastric, lung, and breast cancers, and lymphoma, were similar between incident cases of all age groups and cases restricted to older subjects (≥66 years). Compared with the PPVs for identical cancers in a US Medicare population ≥65 years of age, the PPVs for claims‐based incident cases of gastric and lung cancers, and lymphoma, in patients ≥66 years of age, were higher in Japan (Table S11). In contrast, PPVs for claims‐based algorithms of colorectal and breast cancers were higher for US cases compared with the claims‐based cases in Japan (Table S11). The sensitivity measures of the US claims‐based definitions were consistently higher than the Japanese claims‐based algorithms (Table S11). Comparisons of data from the individual hospitals in Japan with data for US claims‐based definitions are shown in Tables S12 and S13.

## 4 DISCUSSION

Claims databases have been a critical component of real‐world data for decades in the USA, Europe, and other geographic regions for post‐marketing safety surveillance. The use of such data in Japan is in its early stages, but is quickly evolving. A key component in the use of claims data is the demonstration of high validity of the algorithms used to identify study populations and outcomes of interest.

VALIDATE‐J is one of the first and largest multicenter studies conducted in Japan to validate claims‐based algorithms for malignancies specifically developed for the Japanese clinical practice environment. The claims‐based algorithms based on diagnostic and procedural codes identified more than 22 000 cancers of various stages (*in situ* through metastatic), with high specificity and modest‐to‐moderate sensitivity across claims data from two hospitals.

PPV, specificity, and sensitivity were generally high for prevalent cases, and are acceptable algorithms for use in a claims‐based study. PPV and sensitivity were somewhat lower for incident cases with a 12‐month cancer‐free period prior to case ascertainment than for prevalent cases. This may be because the claims‐based algorithms required treatment, and patients were not followed up if they were referred to other centers; patients who were diagnosed, but not treated, at the study hospitals may have been referred to another hospital for more specialized treatment and therefore missed. PPV and sensitivity were lower for melanoma than for other tumor types, due to the very low prevalence of melanoma in Japan, and therefore the claims‐based algorithms for melanoma used here are not suitable for use in further studies. PPV, specificity, and sensitivity measures for all incident cases of colorectal, gastric, lung, and breast cancers, and lymphoma, were similar to validity measures for these same cancers in older patients (≥66 years).

Compared with data from a US validation study using US Medicare claims data,^6^ PPVs for gastric and lung cancers, and lymphoma, were higher using Japanese claims‐based algorithms, while PPVs for colorectal and breast cancers were higher using US claims‐based definitions. Variations in the clinical staging of these cancers between datasets may account for some of these differences. For example, the incidence of Stage IV breast cancer in the Japanese data was higher than expected. For gastric cancers, it is likely that the tumor type affected the validation measures. Differences between countries in the way in which population‐based cancer screening is conducted should also be considered; in Japan, cancer screening is part of the annual health check (which may include chest X‐ray and esophagogastroduodenoscopy) that is covered under universal health insurance. Such an approach may increase the number of false positive results, and this may decrease specificity while maintaining sensitivity. Sensitivity was consistently lower when using the Japanese claims‐based algorithms, and this may relate, in part, to the cancer screening system adopted in Japan.

In one of the first validation studies of claims‐based definitions in Japan, Sato et al assessed the accuracy of 14 definitions for identifying prevalent breast cancer cases using hospital‐based claims at a large teaching hospital in Tokyo.^11^ The optimal definition was derived from combinations of diagnosis and cancer treatment codes (surgery, chemotherapy, medication, radiation procedure), and showed high sensitivity (90.4%), specificity (99.8%), and PPV (87.3%). Compared to the above study which used multiple algorithms, our study only tested one algorithm and reported a nearly identical PPV for breast cancer (86.42%) with a modestly lower sensitivity (67.61%).

The PPVs of the claims‐based algorithms reported here are comparable with those from studies conducted outside Japan, although between‐study comparisons may be limited by differences in the cancer definitions used. For example, validation studies conducted in Europe and Australia using administrative claims data have reported PPV from 58% to 79% and sensitivity from 97% to 99% for incident cases of lung cancer based on diagnostic codes only (*n* = 130),^5^ and PPV/sensitivity of 85%/81% for lung cancers (*n* = 1019) and 91%/95% for colorectal cancers (*n* = 2253) using definitions based on combinations of diagnosis and procedural codes.^18^ PPV/sensitivity of 79%/81%, 88%/72%, and 93%/77%, respectively, for incident cases of lung (*n* = 665), colorectal (*n* = 796), and breast (*n* = 897) cancer was reported using definitions based on hospital discharge codes.^3^ Validation studies conducted in the EU using commercial insurance databases reported PPV of 69% and sensitivity of 66% for lymphoma identified by diagnostic codes (*n* = 340),^4^ and PPV of 82% with negative predictive value of 99%, specificity of 97%, and sensitivity of 91% for the identification of deaths due to breast cancer using algorithms adapted from those previously validated with Canadian data (*n* = 22 413).^19^

Strengths of this study include the large sample size covering multiple tumor types and various disease stages, and the inclusion of data from two large hospitals, both of which had nearly complete cohorts of confirmed cancer cases in the registries. In addition, the claims‐based algorithms were developed by a multidisciplinary team of Japanese experts to ensure relevance to the Japanese clinical practice environment and the use of claims data unique to Japan. Other strengths include additional sensitivity analyses to evaluate incident cases, and the comparison of the Japanese data to similar validation studies conducted in the USA to identify important differences.

A limitation of this study was that the study hospitals were not selected randomly and may not reflect broader Japanese healthcare practices or patient populations. Additionally, the claims data analyzed are derived solely from the study hospitals and, as such, do not represent complete claims data from broader Japanese healthcare systems. The lack of coverage for multiphasic health screening or annual health check‐ups under the Japanese universal health insurance system may have contributed to false negatives, thereby influencing the sensitivity of the analysis. The inclusion of payer‐based claims data would improve the sensitivity of the algorithm, but privacy laws in Japan prohibit the identification of patients directly from administrative healthcare databases, thus making a truly representative validation study not possible. Until privacy restrictions are eased to allow for validation studies to be conducted more easily, researchers must rely on hospital‐based patient sampling of claims data. Despite these existing restrictions, validation studies, such as this study, are steadily increasing and will expand our knowledge about the validity of Japanese claims data for research purposes.

In conclusion, VALIDATE‐J demonstrates that validation of disease‐identifying algorithms for malignancies created specifically for the Japanese clinical practice environment and unique to Japanese claims data is feasible and has high specificity when applied to data from Japanese administrative databases. Data from VALIDATE‐J on disease‐identifying algorithms for RA and infectious diseases will provide additional information on the utility of claims‐based algorithms with Japanese databases. Studies such as VALIDATE‐J will provide researchers with much‐needed knowledge about the validity of Japanese claims data, and may serve as a model for future validation studies in situations where direct identification of patients from administrative healthcare databases is not possible. As with other geographic regions where claims database research is conducted, validation will continue to be a crucial activity to support the integrity of claims database research in Japan.

## CONFLICT OF INTEREST

C.D. and N.S. are employees of Pfizer Inc. T.M., T.H., K.K., S.N., R.H., S.J., M.K. and D.S. have received consultancy fees from Pfizer Inc in connection with this study. H.C. has received funding from Pfizer Inc in connection with this study through her University, and has received grants from the Cystic Fibrosis Foundation, National Institute of Health, and Pfizer Inc. E.N. has no potential conflict of interest to declare. Y.T. has received speaker fees and/or honoraria from AbbVie, Asahi Kasei, Astellas, Bristol‐Myers Squibb, Chugai Pharmaceutical Co., Ltd, Daiichi Sankyo, Eisai, Eli Lilly, Gilead, GSK, Janssen, Mitsubishi Tanabe Pharma, Novartis, Pfizer Inc, Sanofi, and YL Biologics Ltd, and has received research grants from AbbVie, Asahi Kasei, Chugai Pharmaceutical Co., Ltd, Daiichi Sankyo, Eisai, Mitsubishi Tanabe Pharma, and Takeda. S.S. has received consultancy fees from Pfizer Inc in connection with this study, and has received grants from Bristol‐Myers Squibb, Cystic Fibrosis Foundation, Daiichi Sankyo, Janssen, National Institute of Health, PCORI, and Pfizer Inc, and personal consulting fees from Janssen, Medtronic, Merck, and Pfizer Inc.

## AUTHOR CONTRIBUTIONS

All authors participated in the conception and development of this article. H.C., E.N., Y.T., and S.S. were involved in data collection and analysis. T.M., T.H., K.K., S.N., R.H., S.J., M.K., D.S., and Y.T. were involved in data interpretation. All authors critically reviewed the manuscript and approved the final draft prior to submission.

## ETHICS STATEMENT

An Independent Ethics Committee and the Institutional Review Board at each participating hospital approved the study protocol. The study was conducted in accordance with accepted practices for pharmacoepidemiology studies issued by the International Society for Pharmacoepidemiology and the Council for International Organizations of Medical Sciences. Patients identified in the claims databases were not required to provide consent and could opt out from participating in the study.

## Supporting information

**Appendix S1** Supporting information
