# Supplementary material for: Validity of claims‐based algorithms for selected cancers in Japan: Results from the VALIDATE‐J study
Source: Pharmacoepidemiol Drug Saf. 2021 Jun 1;30(9):1153–61. doi: 10.1002/pds.5263 (PMC8453514; doi:10.1002/pds.5263)
Supplement: Supplementary file 1 — Appendix S1 Supporting information [file PDS-30-1153-s001.pdf]

SUPPORTING INFORMATION

TABLE S1 ICD-10 diagnosis codes for the gold standard population of malignancies in the hospital claims databases

Malignancy	ICD-10 codes	Description	Exclusions
All malignancies	C00–C96	Malignant neoplasms	–
	D00–D09	*In situ* neoplasms	–
Breast cancer	C50.x	Malignant neoplasm of breast	–
	D05.x	Carcinoma *in situ* of breast	–
Colorectal cancer	C18.x	Malignant neoplasm of colon	–
	C19.x	Malignant neoplasm of rectosigmoid junction	–
	C20.x	Malignant neoplasm of rectum	–
	D01.0	Carcinoma *in situ* of colon	–
	D01.1	Carcinoma *in situ* of rectosigmoid junction	–
	D01.2	Carcinoma *in situ* of rectum	–
Gastric cancer	C16.x	Malignant neoplasm of stomach	–
	D00.2	Carcinoma *in situ* of stomach	–
Lung cancer	C33	Malignant neoplasm of trachea	–
	C34.x	Malignant neoplasm of bronchus and lung	–
	D02.1	Carcinoma *in situ* of trachea	–
	D02.2	Carcinoma *in situ* of bronchus and lung	–
Lymphoma	C81.x	Hodgkin lymphoma	C88
	C82.x	Follicular lymphoma	.2 Other heavy chain disease
	C83.x	Non-follicular lymphoma	.3 Immunoproliferative small intestinal disease
	C84.x	Mature T/NK-cell lymphomas	.7 Other
	C85.x	Other and unspecified types of non-Hodgkin lymphoma	.9 Unspecified
	C86.x	Other specified types of T/NK-cell lymphoma	C90 Multiple myeloma & malignant plasma cell
	C88	Malignant immunoproliferative diseases	neoplasms
	.0	Waldenström macroglobulinemia	
	.4	MALT-lymphoma	C91
	C91	Lymphoid leukemia	.0 Acute lymphoblastic leukemia
	.1	Chronic lymphocytic leukemia of B-cell type	.7 Other

For Review Only

	.3	Prolymphocytic leukemia of B-cell type	.9 Unspecified
	.4	Hairy-cell leukemia	C92 Myeloid leukemia
	.5	Adult T-cell lymphoma/leukemia (HTLV-1-associated)	C93 Monocytic leukemia
	.6	Prolymphocytic leukemia of T-cell type	C94 Other leukemias of specified cell type
		Mature B-cell leukemia Burkitt-type	
	.8		C95 Leukemia of unspecified cell type
			C96 Other and unspecified malignant neoplasms of lymphoid, hematopoietic and related tissue
Melanoma	C43.x	Malignant melanoma of skin	
	D03.x	Melanoma *in situ*	C44 Other malignant neoplasms of skin
Pancreatic	D01.7	Carcinoma *in situ* of other specified digestive organs (pancreas)	–

Abbreviations: HTLV, human T-lymphotropic virus; ICD-10, International Classification of Diseases, 10th Edition

(https://icd.who.int/browse10/2019/en); MALT, mucosa-associated lymphoid tissue; NK, natural killer.

For Review Only

TABLE S2 Validity measures

**Claims-based algorithms**	**Gold standard diagnosis**[a]	
	+	–
Meets	TP	FP
Does not meet	FN	TN

Abbreviations: FN, false negative; FP, false positive; PPV, positive predictive value; TN, true negative; TP, true positive.

PPV = TP/TP + FP

Specificity = TN/FP + TN

Sensitivity = TP/TP + FN

[a]Confirmation of record in hospital cancer registry.

For Review Only

TABLE S3 Additional demographics and disease characteristics of cases from the cancer registries (both hospitals; prevalent cases)

	**Any malignancy** ***N* = 25 934**	**Colorectal** ***N* = 3519**	**Gastric** ***N* = 3534**	**Lung** ***N* = 2066**	**Breast** ***N* = 4959**	**Pancreatic** ***N* = 582**	**Melanoma** ***N* = 46**	**Lymphoma** ***N* = 1457**
Aged ≥ 65 years, *n* (%)	14 884 (57.4)	2268 (64.5)	2617 (74.1)	1580 (76.5)	1284 (25.9)	438 (75.3)	34 (73.9)	891 (61.2)
UICC stage, *n* (%)								
0 (*in situ*)	2094 (8.1)	193 (5.5)	3 (0.1)	4 (0.2)	1043 (21.0)	4 (0.7)	6 (13.0)	1 (0.1)
I (localized)	8273 (31.9)	622 (17.7)	2339 (66.2)	856 (41.4)	1877 (37.9)	61 (10.5)	9 (19.6)	223 (15.3)
II (regional LN)	4283 (16.5)	672 (19.1)	340 (9.6)	143 (6.9)	1225 (24.7)	105 (18.0)	10 (21.7)	169 (11.6)
III (regional by direct extension)	2577 (9.9)	619 (17.6)	192 (5.4)	303 (14.7)	269 (5.4)	83 (14.3)	1 (2.2)	135 (9.3)
IV (distant)	3070 (11.8)	456 (13.0)	327 (9.3)	510 (24.7)	151 (3.0)	210 (36.1)	1 (2.2)	367 (25.2)
Unknown/missing	5637 (21.7)	957 (27.2)	333 (9.4)	250 (12.1)	394 (7.9)	119 (20.4)	19 (41.3)	562 (38.6)

Abbreviations: LN, lymph node; *N*, total number of patients; *n*, number of patients in each category; UICC, Union for International Cancer Control.

For Review Only

TABLE S4 Demographics and disease characteristics of cases from the cancer registry (Hospital A; prevalent cases)

	**Any malignancy** *N* **= 17 737**	**Colorectal** *N* **= 2334**	**Gastric** *N* **= 2359**	**Lung** *N* **= 1754**	**Breast** *N* **= 4132**	**Pancreatic** *N* **= 352**	**Melanoma** *N* **= 36**	**Lymphoma** *N* **= 803**
Age (years), mean (SD)	64.3 (25.5)	67.9 (11.3)	70.2 (10.3)	69.5 (48.9)	54.7 (12.5)	70.4 (10.5)	69.8 (14.0)	67.1 (13.4)
≥ 65 years, *n* (%)	9802 (55.3)	1502 (64.4)	1732 (73.4)	1331 (75.9)	949 (23.0)	261 (74.1)	25 (69.4)	503 (62.6)
Female, *n* (%)	9352 (52.7)	932 (39.9)	661 (28.0)	589 (33.6)	4132 (100.0)	152 (43.2)	17 (47.2)	366 (45.6)
UICC stage, *n* (%)								
0 (*in situ*)	1798 (10.1)	145 (6.2)	0 (0.0)	3 (0.2)	991 (24.0)	1 (0.3)	6 (16.7)	0 (0.0)
I (localized)	6378 (36.0)	415 (17.8)	1727 (73.2)	829 (47.3)	1623 (39.3)	35 (9.9)	9 (25.0)	170 (21.2)
II (regional LN)	3019 (17.0)	468 (20.1)	215 (9.1)	132 (7.5)	993 (24.0)	80 (22.7)	10 (27.8)	118 (14.7)
III (regional by direct extension)	1751 (9.9)	399 (17.1)	117 (5.0)	286 (16.3)	204 (4.9)	58 (16.5)	1 (2.8)	94 (11.7)
IV (distant)	2046 (11.5)	284 (12.2)	189 (8.0)	421 (24.0)	119 (2.9)	137 (38.9)	1 (2.8)	271 (33.7)
Unknown/missing	2475 (14.0)	623 (26.7)	111 (4.7)	83 (4.7)	202 (4.9)	41 (11.6)	9 (25.0)	150 (18.7)
Method of diagnosis, *n* (%)								
Histology	16 763 (94.5)	2294 (98.3)	2336 (99.0)	1512 (86.2)	4094 (99.1)	262 (74.4)	33 (91.7)	780 (97.1)
Cytology	208 (1.2)	2 (0.1)	4 (0.2)	71 (4.0)	29 (0.7)	6 (1.7)	0 (0)	13 (1.6)
Pathology[a]	2153 (12.1)	277 (11.9)	269 (11.4)	270 (15.4)	492 (11.9)	41 (11.6)	5 (13.9)	97 (12.1)

For Review Only

	Any malignancy *N* = 17 737	Colorectal *N* = 2334	Gastric *N* = 2359	Lung *N* = 1754	Breast *N* = 4132	Pancreatic *N* = 352	Melanoma *N* = 36	Lymphoma *N* = 803
Tumor marker	28 (0.2)	0 (0)	0 (0)	1 (0.1)	0 (0)	0 (0)	0 (0)	0 (0)
Direct visualization	36 (0.2)	9 (0.4)	5 (0.2)	2 (0.1)	0 (0)	5 (1.4)	1 (2.8)	0 (0)
Radiology	636 (3.6)	24 (1.0)	12 (0.5)	166 (9.5)	6 (0.1)	74 (21.0)	1 (2.8)	6 (0.7)
Clinical diagnosis	18 (0.1)	1 (<0.1)	0 (0)	1 (0.1)	0 (0)	2 (0.6)	0 (0)	1 (0.1)
Unknown/missing	41 (0.2)	4 (0.2)	2 (0.1)	0 (0)	2 (<0.1)	3 (0.9)	0 (0)	1 (0.1)
Treatment, *n* (%)								
Surgical	5585 (31.5)	548 (23.5)	747 (31.7)	556 (31.7)	1006 (24.3)	101 (28.7)	26 (72.2)	25 (3.1)
Celoscopic	3936 (22.2)	940 (40.3)	247 (10.5)	219 (12.5)	2315 (56.0)	7 (2.0)	36 (100)	4 (0.5)
Endoscopic	2272 (12.8)	661 (28.3)	1060 (44.9)	1754 (100)	1 (<0.1)	31 (8.8)	36 (100)	2 (0.2)
Radiation therapy	2648 (14.9)	150 (6.4)	7 (0.3)	464 (26.5)	1061 (25.7)	22 (6.3)	1 (2.8)	133 (16.6)
Chemotherapy	4917 (27.7)	689 (29.5)	366 (15.5)	690 (39.3)	1079 (26.1)	170 (48.3)	4 (11.1)	590 (73.5)
Immunotherapy	229 (1.3)	3 (0.1)	2 (0.1)	33 (1.9)	49 (1.2)	1 (0.3)	2 (5.6)	1 (0.1)
Endocrine therapy	398 (2.2)[b]	34 (1.5)	13 (0.6)	75 (4.3)	134 (3.2)[b]	16 (4.5)	36 (100)	8 (1.0)
CCI, mean (SD)[c]	2.1 (1.3)	2.0 (1.3)	2.5 (1.1)	2.4 (1.0)	1.7 (1.0)	2.2 (0.8)	1.9 (1.4)	2.3 (0.8)

Abbreviations: CCI, Charlson Comorbidity Index; LN, lymph node; *N*, total number of patients; *n*, number of patients in each category; SD, standard deviation; UICC, Union for International Cancer Control.

[a]Pathology indicates either histological or cytological diagnoses.

For Review Only

[b]*N* missing = 1.

[c]CCI was calculated by assigning comorbidity scores[1] to patients within each separate cancer pool. The mean score was calculated for each patient by summing their scores and dividing them by the number of scores they had. All patients' average scores were then pooled and divided by the population size of each corresponding cancer category.

For Review Only

TABLE S5 Demographics and disease characteristics of cases from the cancer registry (Hospital B; prevalent cases)

	**Any malignancy** *N* = 8197	**Colorectal** *N* = 1185	**Gastric** *N* = 1175	**Lung** *N* = 312	**Breast** *N* = 827	**Pancreatic** *N* = 230	**Melanoma** *N* = 10	**Lymphoma** *N* = 654
Age (years), mean (SD)	66.0 (14.8)	68.3 (11.1)	70.6 (10.0)	71.9 (9.9)	60.1 (13.3)	71.4 (10.9)	75.2 (14.5)	65.2 (15.9)
≥ 65 years, *n* (%)	5082 (62.0)	766 (64.6)	885 (75.3)	249 (79.8)	335 (40.5)	177 (77.0)	9 (90.0)	388 (59.3)
Female, *n* (%)	3512 (42.8)	459 (38.7)	312 (26.6)	98 (31.4)	827 (100)	108 (47.0)	6 (60.0)	298 (45.6)
UICC stage, *n* (%)								
0 (*in situ*)	296 (3.6)	48 (4.1)	3 (0.3)	1 (0.3)	52 (6.3)	3 (1.3)	0 (0)	1 (0.2)
I (localized)	1895 (23.1)	207 (17.5)	612 (52.1)	27 (8.7)	254 (30.7)	26 (11.3)	0 (0)	53 (8.1)
II (regional LN)	1264 (15.4)	204 (17.2)	125 (10.6)	11 (3.5)	232 (28.1)	25 (10.9)	0 (0)	51 (7.8)
III (regional by direct extension)	826 (10.1)	220 (18.6)	75 (6.4)	17 (5.4)	65 (7.9)	25 (10.9)	0 (0)	41 (6.3)
IV (distant)	1024 (12.5)	172 (14.5)	138 (11.7)	89 (28.5)	32 (3.9)	73 (31.7)	0 (0)	96 (14.7)
Unknown/missing	2892 (35.3)	334 (28.2)	222 (18.9)	167 (53.5)	192 (23.2)	78 (33.9)	10 (100)	412 (63.0)
Method of diagnosis, *n* (%)								
Histology	7206 (87.9)	1114 (94.0)	1145 (97.4)	159 (51.0)	796 (96.3)	87 (37.8)	7 (70.0)	588 (89.9)
Cytology	99 (1.2)	8 (0.7)	2 (0.2)	21 (6.7)	4 (0.5)	12 (5.2)	0 (0)	9 (1.4)
Pathology[a]	281 (3.4)	6 (0.5)	9 (0.8)	65 (20.8)	19 (2.3)	20 (8.7)	0 (0)	33 (5.0)

For Review Only

	Any malignancy *N* = 8197	Colorectal *N* = 1185	Gastric *N* = 1175	Lung *N* = 312	Breast *N* = 827	Pancreatic *N* = 230	Melanoma *N* = 10	Lymphoma *N* = 654
Tumor marker	112 (1.4)	7 (0.6)	1 (<0.1)	8 (2.6)	2 (0.2)	31 (13.5)	0 (0)	4 (0.6)
Direct visualization	415 (5.1)	41 (3.5)	15 (1.3)	49 (15.7)	3 (0.4)	74 (32.2)	3 (30.0)	6 (0.9)
Radiology	3 (<0.1)	1 (<0.1)	0 (0)	1 (0.3)	0 (0)	0 (0)	0 (0)	0 (0)
Clinical diagnosis	0 (0)	0 (0)	0 (0)	0 (0)	0 (0)	0 (0)	0 (0)	0 (0)
Unknown/missing	81 (1.0)	8 (0.7)	3 (0.3)	9 (2.9)	3 (0.4)	6 (2.6)	0 (0)	14 (2.1)
Treatment, *n* (%)								
Surgical	2971 (36.2)	687 (58.0)	411 (35.0)	30 (9.6)	571 (69.0)	59 (25.7)	4 (40.0)	28 (4.3)
Celoscopic	374 (4.6)	142 (12.0)	121 (10.3)	25 (8.0)	6 (0.7)	2 (0.9)	0 (0)	0 (0)
Endoscopic	868 (10.6)	131 (11.1)	359 (30.6)	3 (1.0)	1 (0.1)	27 (11.7)	0 (0)	2 (0.3)
Radiation therapy	750 (9.1)	11 (0.9)	8 (0.7)	25 (8.0)	255 (30.8)	4 (1.7)	0 (0)	31 (4.7)
Chemotherapy	2531 (30.9)	372 (31.4)	236 (20.1)	85 (27.2)	252 (30.5)	98 (42.6)	2 (20.0)	161 (24.6)
Immunotherapy	37 (0.5)	2 (0.2)	2 (0.2)	196 (62.8)	5 (0.6)	0 (0)	0 (0)	0 (0)
N missing	1282	186	236	0	108	52	2	648
Endocrine therapy	964 (11.8)	8 (0.7)	3 (0.3)	3 (1.0)	341 (41.2)	3 (1.3)	0 (0)	6 (0.9)
N missing	1	0	0	0	0	0	0	0
CCI, mean (SD)[b]	2.1 (1.1)	2.2 (1.0)	2.3 (0.9)	2.3 (0.7)	2.0 (0.8)	2.2 (0.8)	2.0 (0.0)	2.2 (0.6)

For Review Only

Abbreviations: CCI, Charlson Comorbidity Index; LN, lymph node; *N*, total number of patients; *n*, number of patients in each category; SD, standard deviation; UICC, Union for International Cancer Control.

[a]Pathology indicates either histological or cytological diagnoses.

[b]CCI was calculated by assigning comorbidity scores[1] to patients within each separate cancer pool. The mean score was calculated for each patient by summing their scores and dividing them by the number of scores they had. All patients' average scores were then pooled and divided by the population size of each corresponding cancer category.

For Review Only

TABLE S6 PPV, specificity, and sensitivity for the alternative algorithm of any malignancy[a] vs gold standard cancer diagnosis

**Cases**	**N_C/N_R**	**PPV, % (95% CI)**	**Specificity, % (95% CI)**	**Sensitivity, % (95% CI)**
Prevalent cases				
Both hospitals	27 645/25 934[b]	73.83 (73.31–74.34)	97.40 (97.34–97.46)	78.70 (78.20–79.19)
Hospital A	17 398/17 737[c]	77.11 (76.48–77.73)	97.50 (97.43–97.58)	75.63 (75.00–76.26)
Hospital B	10 247/8197[d]	68.25 (67.35–69.16)	97.26 (97.17–97.36)	85.32 (84.56–86.09)
Incident cases[e]				
Both hospitals	18 119	66.46 (65.49–67.44)	98.95 (98.91–98.98)	32.97 (32.28–33.65)
Hospital A	11 996	67.36 (66.13–68.59)	99.38 (99.35–99.41)	31.24 (30.41–32.07)
Hospital B	6123	65.00 (63.40–66.60)	99.60 (99.58–99.62)	36.34 (35.13–37.54)

Abbreviations: CI, confidence interval; ICD-10, International Classification of Diseases, 10th Edition; N_C, number of claims-based cases; N_R, number of registry cases; *n*, number of duplicate cases; PPV, positive predictive value.

[a]Identified using two cancer diagnoses having the same first three digits of the ICD-10 codes, within the same claim-month or ±1 claim-month.

For Review Only

Number of duplicate cases removed for the analysis: [b]n = 2901; [c]n = 2190; [d]n = 711.

[e]Incident cases represent those with a 12-month cancer-free period and with claims and registry period in the same month.

For Review Only

TABLE S7 PPV, specificity, and sensitivity of the claims-based algorithms for selected malignancies vs gold standard cancer diagnosis (Hospital A; prevalent cases)

**Malignancy**	**N_C/N_R**	**PPV, % (95% CI)**	**Specificity, % (95% CI)**	**Sensitivity, % (95% CI)**
Any malignancy	14 257/17 737[a]	82.81 (82.19–83.43)	98.46 (98.40–98.52)	66.56 (65.87–67.26)
Colorectal	878/2334[b]	89.18 (87.13–91.23)	99.95 (99.93–99.96)	33.55 (31.63–35.46)
Gastric	978/2359[c]	93.05 (91.45–94.64)	99.96 (99.95–99.97)	38.58 (36.61–40.54)
Lung	968/1754[d]	90.29 (88.42–92.15)	99.95 (99.94–99.96)	49.83 (47.49–52.17)
Breast	3172/4132[e]	88.27 (87.15–89.39)	99.79 (99.76–99.81)	67.76 (66.34–69.19)
Pancreatic	193/352	90.67 (86.57–94.78)	99.99 (99.99–99.99)	49.72 (44.49–54.94)
Melanoma	30/36	46.67 (28.81–64.52)	99.99 (99.99–100.00)	38.89 (22.96–54.81)
Lymphoma	430/803[f]	85.12 (81.75–88.48)	99.96 (99.95–99.97)	45.58 (42.13–49.02)

For Review Only

Abbreviations: CI, confidence interval; N_C, number of claims-based cases; N_R, number of registry cases; *n*, number of duplicate cases;

PPV, positive predictive value.

Number of duplicate cases removed for this analysis: [a]*n* = 2190; [b]*n* = 167; [c]*n* = 165; [d]*n* = 75; [e]*n* = 220 plus 8 males; [f]*n* = 8.

For Review Only

TABLE S8 PPV, specificity, and sensitivity of the claims-based algorithms for selected malignancies vs gold standard cancer diagnosis (Hospital B; prevalent cases)

**Malignancy**	**N_C/N_R**	**PPV, % (95% CI)**	**Specificity, % (95% CI)**	**Sensitivity, % (95% CI)**
Any malignancy	7851/8197[a]	71.94 (70.95–72.93)	98.15 (98.07–98.22)	68.90 (67.90–69.91)
Colorectal	839/1185[b]	79.38 (76.64–82.12)	99.86 (99.84–99.88)	56.20 (53.38–59.03)
Gastric	806/1175[c]	80.65 (77.92–83.37)	99.88 (99.86–99.90)	55.32 (52.48–58.16)
Lung	167/312[d]	75.45 (68.92–81.98)	99.97 (99.96–99.98)	40.38 (34.94–45.83)
Breast	708/827[e]	78.11 (75.06–81.15)	99.88 (99.86–99.90)	66.87 (63.66–70.08)
Pancreatic	110/230	80.91 (73.56–88.25)	99.98 (99.98–99.99)	38.70 (32.40–44.99)
Melanoma	7/10	57.14 (20.48–93.80)	100.00 (99.99–100.00)	40.00 (9.64–70.36)
Lymphoma	535/654[f]	82.43 (79.21–85.65)	99.93 (99.91–99.94)	67.43 (63.84–71.02)

For Review Only

Abbreviations: CI, confidence interval; N_C, number of claims-based cases; N_R, number of registry cases; *n*, number of duplicate cases; PPV, positive predictive value.

Number of duplicate cases removed for this analysis: [a]*n* = 711; [b]*n* = 60; [c]*n* = 40; [d]*n* = 4; [e]*n* = 6 plus 29 males; [f]*n* = 2.

For Review Only

TABLE S9 PPV, sensitivity, and specificity of the claims-based algorithms for selected malignancies vs gold standard cancer diagnosis (Hospital A; incident cases[a])

Malignancy	N_R	PPV, % (95% CI)	Specificity, % (95% CI)	Sensitivity, % (95% CI)
Any malignancy	11 996	71.88 (70.46–73.29)	99.63 (99.61–99.65)	23.16 (22.40–23.91)
Colorectal	1470	68.83 (64.20–73.46)	99.96 (99.95–99.97)	18.03 (16.06–19.99)
Gastric	1435	82.21 (78.45–85.96)	99.98 (99.97–99.98)	22.86 (20.68–25.03)
Lung	1344	81.17 (77.89–84.45)	99.97 (99.96–99.97)	33.04 (30.52–35.55)
Breast	2603	64.30 (60.24–68.36)	99.94 (99.93–99.95)	13.22 (11.91–14.52)
Pancreatic	307	85.71 (78.52–92.90)	100.00 (99.99–100.00)	25.41 (20.54–30.28)
Melanoma	28	38.89 (16.37–61.41)	100.00 (99.99–100.00)	25.00 (8.96–41.04)
Lymphoma	558	74.88 (69.09–80.68)	99.98 (99.98–99.99)	28.85 (25.09–32.61)

Abbreviations: CI, confidence interval; N_R, number of registry cases; PPV, positive predictive value.

[a]Incident cases represent those with a 12-month cancer-free period and with claims and registry period in the same month.

For Review Only

TABLE S10 PPV, sensitivity, and specificity of the claims-based algorithms for selected malignancies vs gold standard cancer diagnosis (Hospital B; incident cases[a])

Malignancy	N_R	PPV, % (95% CI)	Specificity, % (95% CI)	Sensitivity, % (95% CI)
Any malignancy	6123	75.49 (73.57–77.41)	99.84 (99.83–99.86)	23.84 (22.78–24.91)
Colorectal	870	61.90 (56.71–67.10)	99.96 (99.95–99.97)	23.91 (21.07–26.74)
Gastric	884	68.48 (62.80–74.16)	99.97 (99.97–99.98)	20.85 (18.11–23.59)
Lung	292	71.43 (61.77–81.09)	100.00 (100.00–100.00)	20.55 (15.91–25.18)
Breast	582	41.86 (33.35–50.37)	99.98 (99.97–99.98)	9.28 (6.92–11.64)
Pancreatic	201	71.15 (58.84–83.47)	100.00 (99.99–100.00)	18.41 (13.05–23.77)
Melanoma	8	66.67 (34.32–99.99)	100.00 (100.00–100.00)	25.00 (5.01–55.01)
Lymphoma	477	80.79 (75.37–86.21)	99.99 (99.98–99.99)	34.38 (30.12–38.64)

Abbreviations: CI, confidence interval; N_R, number of registry cases; PPV, positive predictive value.

[a]Incident cases represent those with a 12-month cancer-free period and with claims and registry period in the same month.

For Review Only

TABLE S11 PPV, specificity, and sensitivity for claims-based cases in Japan (both hospitals; incident cases[a] aged ≥ 66 years) and US Medicare population[b,c]

**Malignancy**	**N_R**	**PPV (%) (95% CI)**	**Specificity (%) (95% CI)**	**Sensitivity (%) (95% CI)**
**Colorectal**				
Japan	1493	63.17 (58.79–67.54)	99.94 (99.93–99.95)	19.76 (17.74–21.78)
US	2017	70.95	99.62	67.25
**Gastric**				
Japan	1689	78.20 (74.58–81.82)	99.96 (99.96–99.97)	23.15 (21.14–25.16)
US	276	59.78	99.93	69.92
**Lung**				
Japan	1228	80.88 (77.35–84.41)	99.97 (99.96–99.98)	31.35 (28.76–33.95)
US	1344	75.89	99.79	56.35
**Breast**				
Japan	787	56.46 (49.74–63.18)	99.97 (99.96–99.98)	14.99 (12.50–17.49)
US	1150	81.74	99.84	46.91
**Lymphoma**				
Japan	636	75.85 (70.70–81.00)	99.98 (99.97–99.98)	31.60 (27.99–35.22)
US	564	61.52	99.86	55.17

For Review Only

Abbreviations: CI, confidence interval; N_R, number of registry cases; PPV, positive predictive value.

[a]Incident cases represent those with a 12-month cancer-free period and with claims and registry period in the same month.

[b]Aged ≥ 65 years with ≥ 1 cancer diagnosis; 12-month cancer-free period (1997–2000).

[c]US data adapted from Setoguchi et al.[2]

For Review Only

TABLE S12 PPV, specificity, and sensitivity for claims-based cases in Japan (Hospital A; incident cases[a]) and US Medicare population[b,c]

Malignancy	N_R	PPV, % (95% CI)	Specificity, % (95% CI)	Sensitivity, % (95% CI)
**Colorectal**				
Japan	1470	68.83 (64.20–73.46)	99.96 (99.95–99.97)	18.03 (16.06–19.99)
US	2017	70.95	99.62	67.25
**Gastric**				
Japan	1435	82.21 (78.45–85.96)	99.98 (99.97–99.98)	22.86 (20.68–25.03)
US	276	59.78	99.93	69.92
**Lung**				
Japan	1344	81.17 (77.89–84.45)	99.97 (99.96–99.97)	33.04 (30.52–35.55)
US	1344	75.89	99.79	56.35
**Breast**				
Japan	2603	64.30 (60.24–68.36)	99.94 (99.93–99.95)	13.22 (11.91–14.52)
US	1150	81.74	99.84	46.91
**Lymphoma**				
Japan	558	74.88 (69.09–80.68)	99.98 (99.98–99.99)	28.85 (25.09; 32.61)
US	564	61.52	99.86	55.17

Abbreviations: CI, confidence interval; N_R, number of registry cases; PPV, positive predictive value.

[a]Incident cases represent those aged ≥ 66 years with a 12-month cancer-free period and with claims and registry period in the same month.

For Review Only

[b]Aged ≥ 65 years with ≥ 1 cancer diagnosis; 12-month cancer-free period (1997–2000).

[c]US data adapted from Setoguchi et al.[2]

For Review Only

TABLE S13 PPV, specificity, and sensitivity for claims-based cases in Japan (Hospital B; incident cases[a]) and US Medicare population[b,c]

Malignancy	N_R	PPV, % (95% CI)	Specificity, % (95% CI)	Sensitivity, % (95% CI)
**Colorectal**				
Japan	870	61.90 (56.71–67.10)	99.96 (99.95–99.97)	23.91 (21.07–26.74)
US	2017	70.95	99.62	67.25
**Gastric**				
Japan	884	68.48 (62.80–74.16)	99.97 (99.97–99.98)	20.85 (18.11–23.59)
US	276	59.78	99.93	69.92
**Lung**				
Japan	272	71.43 (61.77–81.09)	100.00 (100.00–100.00)	20.55 (15.91–25.18)
US	1344	75.89	99.79	56.35
**Breast**				
Japan	582	41.86 (33.35–50.37)	99.98 (99.97–99.98)	9.28 (6.92–11.64)
US	1150	81.74	99.84	46.91
**Lymphoma**				
Japan	558	74.88 (69.09–80.68)	99.98 (99.98–99.99)	28.85 (25.09–32.61)
US	564	61.52	99.86	55.17

Abbreviations: CI, confidence interval; N_R, number of registry cases; PPV, positive predictive value.

[a]Incident cases represent those aged ≥ 66 years with a 12-month cancer-free period and with claims and registry period in the same month.

For Review Only

[b]Aged ≥ 65 years with ≥ 1 cancer diagnosis; 12-month cancer-free period (1997–2000).

[c]US data adapted from Setoguchi et al.[2]

For Review Only

REFERENCES

1. Charlson ME, Pompei P, Ales KL, MacKenzie CR. A new method of classifying prognostic comorbidity in longitudinal studies: development and validation. *J Chronic Dis* 1987;40:373-383.

2. Setoguchi S, Solomon DH, Glynn RJ, Cook EF, Levin R, Schneeweiss S. Agreement of diagnosis and its date for hematologic malignancies and solid tumors between medicare claims and cancer registry data. *Cancer Causes Control* 2007;18:561-569.

For Review Only
